# Stereotactic body radiation therapy for the primary treatment of localized prostate cancer

**DOI:** 10.1007/s13566-012-0067-2

**Published:** 2012-09-12

**Authors:** Caspian Oliai, Rachelle Lanciano, Brian Sprandio, Jun Yang, John Lamond, Steven Arrigo, Michael Good, Michael Mooreville, Bruce Garber, Luther W. Brady

**Affiliations:** 1 Drexel University College of Medicine/Hahnemann University Hospital, 230 N Broad St, Philadelphia, PA 19102 USA; 2 Philadelphia CyberKnife Center, 2010 West Chest Pike Suite 115, Havertown, PA 19083 USA

**Keywords:** Stereotactic body radiation therapy, Prostate cancer, Hypofractionation, Alpha/beta ratio, Dose escalation

## Abstract

**Objective:**

The low alpha/beta ratio of prostate cancer suggests that hypofractionated schemes of dose-escalated radiotherapy should be advantageous. We report our experience using stereotactic body radiation therapy (SBRT) for the primary treatment of prostate cancer to assess efficacy and toxicity.

**Methods:**

From 2007 to 2010, 70 patients (51 % low risk, 31 % intermediate risk, and 17 % high risk) with localized prostate cancer were treated with SBRT using the CyberKnife system. One-third of patients received androgen deprivation therapy. Doses of 37.5 Gy (*n* = 29), 36.25 Gy (*n* = 36), and 35 Gy (*n* = 5) were administered in five fractions and analyzed as high dose (37.5 Gy) vs. low dose (36.25 and 35 Gy).

**Results:**

At a median 27 and 37 months follow-up, the low and high dose groups' median PSA nadir to date was 0.3 and 0.2 ng/ml, respectively. The 3-year freedom from biochemical failure (FFBF) was 100 %, 95.0 % and 77.1 % for the low-, intermediate- and high-risk patients. A dose response was observed in intermediate- and high-risk patients with 72 % vs. 100 % 3-year FFBF for the low and high dose groups, respectively (*p* = 0.0363). Grade III genitourinary toxicities included 4 % acute and 3 % late (all high dose). Potency was preserved in 83 % of hormone naïve patients.

**Conclusion:**

CyberKnife dose escalated SBRT for low-, intermediate- and high-risk prostate cancer exhibits favorable efficacy with acceptable toxicity.

## Introduction

Technological and radiobiological advances in early-stage prostate cancer treatment have led to a debate within the radiation oncology community over the optimal treatment. Long-term results from prospective [1, 2] and randomized dose escalation trials [3–5], comparing doses of 74–81 Gy to doses <70 Gy show dose escalation with conventional fractionation (1.8–2.0 Gy per fraction) improves freedom from biochemical failure (FFBF) and disease progression with acceptable toxicity. However, despite dose escalation, improvements are needed to consistently achieve the highest outcomes.

Brenner and Hall [6] suggested hypofractionation for prostate cancer as an alternative radiation scheme based on radiobiological modeling from external beam radiation therapy (EBRT) and low-dose rate brachytherapy series. They estimated that the alpha/beta (α/β) ratio for prostate cancer was uniquely low at 1.5 Gy as opposed to about 10 Gy for other cancers. An α/β ratio <2 Gy would be clinically significant since late-responding normal tissue near the prostate has an α/β ratio between 2 and 4 Gy. Therefore, a hypofractionated dose scheme has the potential to produce a more advantageous therapeutic ratio. The low α/β ratio for prostate cancer is supported by Fowler et al. [7], who suggested that it may be as low or even lower than 1.5 Gy. Trials designed to assess these estimates showed that moderately hypofractionated doses were superior to conventional fractionation with respect to biochemical control and survival with acceptable levels of toxicity [8–10]. Indeed, reviews of clinical datasets support a mean α/β ratio <1.5 Gy [11, 12].

High dose-rate (HDR) brachytherapy has shown excellent hypofractionated monotherapy results [13], however, limitations on its use include clinical experience, technology availability and patient participation in an invasive procedure. Stereotactic body radiotherapy (SBRT), typically five fractions, is also well suited for hypofractionated radiation delivery. Several recent SBRT publications report promising clinical efficacy with minimal toxicity, including series with 5 years follow-up [14–22]. Furthermore, possible reduced cost and patient convenience make SBRT a desirable treatment modality. We report on intermediate-term toxicity and efficacy of our SBRT experience for early stage prostate cancer.

## Methods

Eighty-three early stage prostate cancer patients received SBRT at the Philadelphia CyberKnife Center from 2007 through 2010. Patients with ≥12 months of follow-up are included in this IRB approved retrospective analysis. SBRT was delivered using the CyberKnife (Accuray Inc., Sunnyvale, CA) with MultiPlan inverse treatment planning and motion tracking of internal fiducials. Treatment planning began with transrectal or transperineal ultrasound-guided placement of four gold fiducials into the prostate. A CT scan (1.25-mm slice thickness) was obtained 10–14 days later to allow inflammation to subside and ensure fiducials did not migrate. T2 fast echo MRI was obtained and three-dimensionally registered by fiducials to the CT in the majority of patients.

**Fig. 1:**
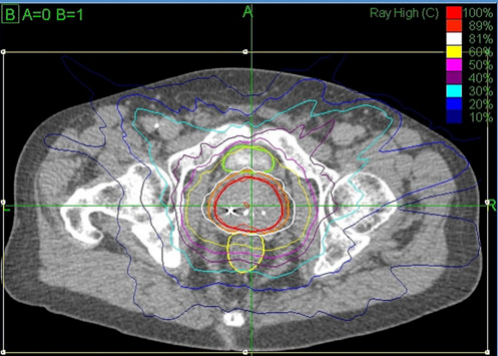
CyberKnife SBRT treatment plan (36.25 Gy × 5 fractions prescribed to the 81 % isodose line (*white*) with a 44.75 Gy maximum dose. Shown are the prostate (43.6 cm^3^, *red*) and PTV (77.8 cm^3^, *orange*). A bladder (*green*) volume of 1.66 cm^3^ received 37 Gy and 0.4 cm^3^ of the rectum (*yellow*) received 36 Gy

The prostate, seminal vesicles, rectum, bladder, penile bulb, and bowel were contoured (Fig. 1). Urethra contouring was preferred, but not required. The clinical target volume (CTV) was the prostate for low-risk patients and the prostate plus 2-cm seminal vesicle base for four intermediate-risk and two high-risk patients. All of the other intermediate- and high-risk patients had a CTV which encompassed the prostate without the seminal vesicle bases. A total dose of 35, 36.25 or 37.5 Gy, delivered in five fractions, was prescribed to the planning target volume (PTV) that consisted of the CTV with a 5-mm margin in all directions except 3 mm posteriorly. Dose administered was standard throughout our center, which was based on published data available at that time. Initially, we treated patients with 35 Gy, followed by 37.5 Gy, and at the time of this publication to 36.25 Gy, which began when we participated in an Accuray Inc study. The dosimetric goal was to cover at least 95 % of the PTV with the prescribed dose normalized to the 75–85 % isodose line (dose heterogeneity 17–33 %). Less than 1 cm^3^ of rectum received 36 Gy, 50 % of the prescribed dose could not cross the posterior rectal wall, and <10 cm^3^ of bladder received 37 Gy. The average CTV and PTV were 55.0 cm^3^ (std dev 27.5 cm^3^) and 95.4 cm^3^ (std dev 41.3 cm^3^), respectively.

Orthogonal 120-kV X-ray image pairs were obtained throughout treatment for use in motion tracking. The real-time prostate position was locked-on by the relative fiducial position on the X-rays. For those patients with evenly distributed fiducials in the prostate quadrants, the prostate’s rotation was also tracked and corrections were made in real time.

PSA nadir to date is defined as the lowest PSA value following SBRT. Benign PSA bounce was defined as a PSA rise of ≥0.2 ng/ml above its previous nadir with subsequent decline to that nadir or lower. Biochemical failure (BF) was assessed using the nadir + 2 (Phoenix) definition. Toxicity was assessed using the Radiation Therapy Oncology Group criteria; acute toxicity occurred within 3 months and late toxicity >3 months following treatment. Risk group was assessed with the AUA system. Unpaired *t*-tests assessed statistical significance.

## Results

### Patient and treatment characteristics

Seventy patients with a median 31 months (range 13–51) follow-up were analyzed (Table 1) including one patient who died from causes other than prostate cancer or treatment 34 months after SBRT. Neoadjuvant androgen deprivation therapy (ADT) in the form of luteinizing hormone-releasing hormone agonist was used in 33 % of patients. The use of ADT was dependent on individual urologist and radiation oncologist preference, however, the percentage of the intermediate- and high-risk patients receiving hormones in the neoadjuvant and monotherapy groups were similar. Selected low-risk patients received ADT to shrink the prostate before SBRT. Eight patients received ADT for ≥24 months, 13 for 6–24 months, and 2 for <6 months. Patients treated with SBRT plus ADT are termed the “neoadjuvant group” and those treated with SBRT exclusively the “monotherapy group.” Patients who received 35 Gy or 36.25 Gy are termed the “low dose group” and those who received 37.5 Gy the “high-dose group.” Tumor stage, Gleason score, risk group, consecutive treatment days and ADT usage were not significantly different between dose groups.

**Table 1:** Patient and tumor characteristics

	All patients	High dose	Low dose
Number	70	29	41
Dose (*n*)			
35 Gy	5	0	5
36.25 Gy	36	0	36
37.5 Gy	29	29	0
Age (years)			
Median (range)	68 (46–88)	67 (46–80)	68 (51–88)
CTV (cm^3^)			
Median (range)	47.5 (19.0–162.2)	49.87 (31.6–162.2)	44.4 (19.0–107.9)
iPSA (ng/ml)			
Median (range)	5.6 (1.1–39.4)	5.7 (1.1–29.5)	5.6 (1.6–39.4)
Tumor stage (*n*/%)			
T1b	1	0/0 %	1/2 %
T1c	52	21/73 %	31/77 %
T2a	7	5/17 %	2/4 %
T2b	4	3/10 %	1/2 %
T2c	6	0/0 %	6/15 %
Gleason score (*n*/%)			
5	2	1/3 %	1/2 %
6	39	15/52 %	24/59 %
7	23	10/35 %	13/32 %
8	6	3/10 %	3/7 %
Risk group (*n*/%)			
Low	36/51 %	14/48 %	23/56 %
Intermediate	22/31 %	11/38 %	10/24 %
High	12/17 %	4/14 %	8/20 %
Maximum baseline GU toxicity grade (*n*/%)			
I	36/51 %	16/55 %	20/49 %
II	9/13 %	3/10 %	6/15 %
Treatment groups (*n*/%)			
Monotherapy	47/67 %	20/69 %	27/66 %
Neoadjuvant	23/33 %	9/31 %	14/34 %
Consecutive treatment days (Mon–Fri) (*n*/%)			
Consecutive days	12/17 %	4/14 %	8/20 %

Twenty-nine patients (41 %) received 37.5 Gy, 36 patients (51 %) received 36.25 Gy, and five patients (7 %) received 35 Gy. Most patients received their treatment over 7 days (45 %), with the next highest proportion receiving treatment over 5 days (17 %), followed by those on treatment for 8 days (12 %). The remainder completed treatment between 9 and 15 days with one non-compliant patient receiving his final fraction several weeks later, all due to poor adherence to their schedule. Treatment was delivered with an average of 187 (SD = 43.0) non-coplanar beams. X-ray images were taken every three to five beams to track the prostate’s movement.

### PSA response and biochemical control

**Fig. 2:**
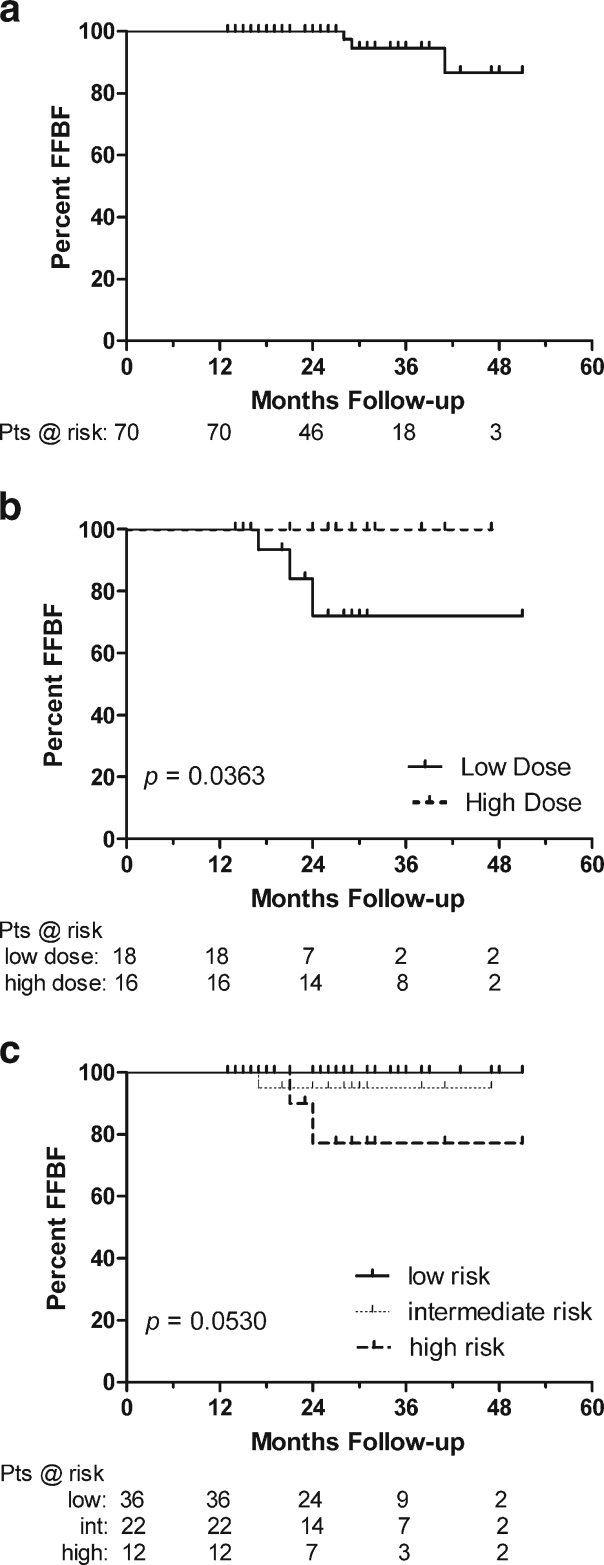
FFBF for **a** all patients, **b** intermediate- and high-risk patients by dose group and **c** all patients by risk group

PSA values began to gradually decline shortly after SBRT. The low-dose and high-dose groups’ median PSA nadirs to date were 0.3 and 0.2 ng/ml, respectively (Table 2). Three patients (4.2 %), all in the low dose group (Table 3), experienced BF; two were high-risk and one intermediate-risk, despite the shorter median follow-up in the low-dose group (27 vs. 37 months). Overall 3-year actuarial FFBF was 94.5 % (Fig. 2a). A dose response was observed for the intermediate- and high-risk patients (*p* = 0.0363) with 3-year actuarial FFBF rates of 72 % for the low dose group and 100 % for the high dose group (Fig. 2b). Inclusion of low-risk patients showed a dose response trend (*p* = 0.0775). Three-year FFBF rates were 100 %, 95.0 % and 77.1 % for the low-, intermediate- and high-risk groups, respectively (*p* = 0.0530) (Fig. 2c). Neoadjuvant ADT in the high-, intermediate, and low-risk groups was used in 58 %, 50 %, and 14 % of patients, respectively.

**Table 2:** PSA nadir for patients in the monotherapy group without biochemical failure separated by risk group

	≤0.1 ng/ml	0.1 < *x* ≤ 0.5 ng/ml	0.5 < *x* ≤ 1.0 ng/ml	1.0 < *x* ≤ 1.5 ng/ml	>1.5 ng/ml
Monotherapy, n = 47	8/17 %	25/53 %	7/15 %	2/4 %	5/11 %
Low, *n* = 32	6/19 %	15/47 %	6/19 %	2/6 %	3/9 %
Int, *n* = 10	1/10 %	6/60 %	1/10 %		2/20 %
High, *n* = 5	1/20 %	4/80 %

**Table 3:** PSA response and toxicity by dose group

Dose group	Median follow-up (months)	Biochemical Failure (n)	Median PSA nadir to date (ng/ml)	Late GU toxicity	
				Grade II	Grade III
High dose, *n* = 29	37 (24–48)	0	0.2	7/24 %	2/7 %
Low dose, *n* = 41	27 (13–51)	3	0.43	13/32 %	0

In the monotherapy group (median follow-up 29 months), the two patients with BF (one high- and one intermediate-risk; dose 36.25 Gy) experienced time to biochemical failure (TTBF) at 24 and 17 months. One had a bone scan suggestive of metastatic spread and received 8 Gy × 3 fractions to the right ischium. The monotherapy group also includes five patients (9 %) with a benign PSA bounce at a median time of 19 months (range 16–20) after SBRT. Of those patients without BF, the median PSA nadir was 0.4 ng/ml (range 0.05–2.9 ng/ml). The largest proportion of PSA nadirs were >0.1 but ≤0.5 ng/ml (Table 2). Median PSA at most recent follow-up in the monotherapy group patients who did not experience BF (*n* = 45) was 0.4 ng/ml.

In the neoadjuvant group, the sole failure occurred 21 months following SBRT with metastasis to the thoracic spine. This high-risk patient received ADT >24 months and a SBRT dose of 35 Gy. Subsequently, this patient was treated with additional ADT, chemotherapy, and palliative radiotherapy. Ninety-one percent of PSA nadirs were <0.1 ng/ml, influenced heavily by hormonal effects. No benign PSA bounces occurred.

### Toxicity

Acute genitourinary (GU) toxicity was moderate and rates were similar in each dose group (Table 4). Acute grade II GU toxicities were seen in 19 % (*n* = 13), of which nine patients were in the low dose group and 4 were in the high dose group. Acute grade III GU toxicities were observed in 4 % (*n* = 3), of which two patients were in the low dose group and one patient was in the high dose group. Two of the three grade III acute GU toxicities were frequency at least every hour and one was gross hematuria. These toxicities resolved within 3 months of treatment. However, one case of frequency at least every hour reappeared as a late toxicity. Acute gastrointestinal (GI) toxicity was limited to 4 % grade II toxicity.

**Table 4:** Acute and late maximum toxicity assessed by the RTOG toxicity scale

	Grade I	Grade II	Grade III
Acute toxicity			
GU	39/56 %	13/19 %	3/4 %
Low dose	22/54 %	9/22 %	2/5 %
High dose	17/59 %	4/14 %	1/3 %
GI	12/17 %	3/4 %	0 %
Low dose	8/20 %	3/7 %	
High dose	4/14 %		
Late toxicity			
GU	31/44 %	20/29 %	2/3 %
Low dose	17/41 %	13/32 %	
High dose	14/48 %	7/24 %	2/7 %
GI	7/10 %	6/9 %	0 %
Low dose	4/10 %	4/10 %	
High dose	3/10 %	2/7 %	
ED^a^	8/28 %	5/17 %	0 %
Low dose	4/25 %	3/19 %	
High dose	4/31 %	2/15 %	

^a^ED assessed by CTCAE v3

Late toxicity rates were acceptable with severe grade III toxicity occurring in only two patients (Table 4). Late grade II GU toxicities were observed in 29 % (*n* = 20), of which 13 patients were in the low dose group and seven were in the high dose group. Late grade III GU toxicities were observed in 3 % (*n* = 2), of which both patients were in the high dose group. One patient with a 162 cm^3^ CTV (the largest prostate in our patient sample) had acute grade III frequency that resolved within 2 weeks of alpha-blocker and prophylactic antibiotic treatment, but recurred 6 months later. His symptoms improved to grade II immediately after trans-urethral resection of the prostate (TURP) 13 months after SBRT. Pathology of the resected tissue was negative for tumor. The second patient with late grade III GU toxicity experienced urinary retention. He had benign prostate hypertrophy (BPH) and grade II symptoms at baseline. At 14 months following SBRT, his symptoms progressed to grade III then completely subsided following laser TURP. Late GI toxicity was limited to 4 % grade II toxicity. At most recent follow-up, no patient was experiencing grade III or higher toxicity. The most severe persistent toxicities are grade II GU (19 %) and GI (4 %) with no difference by dose group.

Erectile dysfunction (ED) was assessed for the 29 monotherapy patients who were potent before SBRT. At last follow-up, 17 % lost the ability to achieve erections strong enough for penetration and required ED medication for intercourse. No monotherapy patient who was potent before SBRT developed ED refractory to medical treatment.

## Discussion

The 3-year actuarial FFBF rates of 100 %, 95.0 % and 77.1 % for low-, intermediate- and high-risk groups and overall toxicity are concordant with published SBRT outcomes (Table 5). We further analyzed patients separately as those who received neoadjuvant ADT and those who received SBRT monotherapy due to the confounding effect that hormonal therapy can have on obscuring true PSA decline and BF rates. While our study lacks the long-term follow-up essential to make conclusions regarding rates of BF, it does offer sufficient follow-up to evaluate nadir PSA to date.

**Table 5:** SBRT publications for primary treatment of prostate cancer

Author	Dose scheme	No. of patients	Risk group	Median follow-up (mo)	Freedom from biochemical failure (by risk)	Late grade III GU/GI Toxicity	Patients receiving ADT
Oliai et al. 2012	7.0, 7.25 and 7.5 Gy × 5	70	low, int, high	31	100 %, 94.7 %, 77.1 % 3-year	3 %/0 %	33 %
King et al. 2012	7.25 Gy × 5	67	low	32	94 % 4-year	3 %/0 %	none
McBride et al. 2011	7.25 and 7.5 Gy × 5	45	low	44	97.7 % 3-year	2 %/5 %	none (within 6 months of treatment)
Boike et al. 2011	9, 9.5, and 10 Gy × 5	45	low, int	30/18/12 by dose	100 %	4 %/2 % (Gr 4)	22 %
Kang et al. 2011	8, 8.5, and 9 Gy × 4	44	low, int, high	40	100 %, 100 %, 90.8 %	0 %/0 %	87 %
Freeman et al. 2011	7 and 7.25 Gy × 5	41	low	60	92.7 % 5-year	2.5 %/0 %	none
Bolzicco et al. 2010	7 Gy × 5	45	low, int	20	100 %	2.2 %/0 %	38 %
Katz et al. 2010	7 and 7.25 Gy × 5	304	low, int, high	30	99 %, 84 %, 100 %	<1 %/0 %	19 %
Friedland et al. 2009	7 and 7.25 Gy × 5	112	low, int, high	24	97.3 %	0 %/<1 %	19 %
Madsen et al. 2007	6.7 Gy × 5	40	low	41	90 % 4-year	0 %/0 %	none

Zelefsky et al. [23] showed that 24-month nadir PSA was predictive of distant recurrence. They reported that patients who fail to achieve a nadir PSA <1.5 ng/ml within 24 months have a higher propensity for relapsing disease. Lamb et al. [24] further support that the nadir PSA is the strongest prognostic indicator of all early PSA-based indicators with the ability to predict long-term survival. We regard the low nadir PSA in our series as indicative of a favorable outcome despite the limited follow-up. For example, our monotherapy data show 86 % of patients with >24 months follow-up reached a nadir <1.5 ng/ml before 24 months post-SBRT. We report the nadir PSA to date of the high dose group to be less than that of the low dose group (0.2 vs. 0.3 ng/ml). It is important to note that the median follow-up for the high dose group is 10 months greater than the low dose group. Therefore, the patients receiving high dose had additional time for their PSA to fall.

It is worth commenting on the median PSA at most recent follow-up, despite the relatively slow-growing nature of prostate cancer. At a median follow-up of 32 months, King et al. report a low median PSA at most recent follow-up of 0.5 ng/ml in low-risk patients who did not receive ADT. Our results are similar in the monotherapy group (low-, intermediate-, high-risk patients), which have a median PSA at most recent follow-up of 0.4 ng/ml at a median follow-up of 29 months. Although the patients have several more years to experience BF, these PSA values have reached low levels in a short duration, which may be an indication of favorable long-term control.

Figure 2b represents a dose response in intermediate- and high-risk groups, which had similar proportion of patients receiving neoadjuvant ADT in each group (50 % vs. 58 %). Few other SBRT publications [16, 17] provide outcomes stratified by intermediate- and high-risk. In the case of IMRT, Zelefsky et al. report an 8-year FFBF rate of 67 % for high-risk patients receiving 81 Gy IMRT with conventional fractionation. While this is a longer follow-up than the current study, it is important to note that the decline in FFBF between 3 and 8 years was modest (approximately 77 % vs. 67 %), most likely due to the tendency of early failure for high-risk prostate cancer patients [25]. Thus, the current report’s observed 77 % 3-year FFBF rate for high-risk patients suggests SBRT may have a role in the treatment of these patients. In regard to intermediate-risk patients treated with SBRT, Katz et al. has presented data with 4-year actuarial FFBF of 91 % in those with intermediate risk disease treated with 35–36.25 Gy plus 28 % receiving ADT. The rate of late grade III GU toxicity was 1.2 % and median PSA at 46 month follow-up was 0.1 ng/ml [26]. Furthermore, randomized conventionally fractionated dose-escalation trials have shown dose responses for intermediate- and high-risk patients [4, 27] similar to those observed here, but limited SBRT dose escalation results exist.

Katz et al. [28] reported comparable efficacy in primarily low-risk patients treated with 35 and 36.25 Gy. Their results indicate a trend toward higher late GU toxicity as dose increases, which is also a trend in our study with respect to late grade III GU toxicity but not grade II toxicity (Table 4). Boike et al. [21] report dose escalation of 45, 47.5 and 50 Gy for low- and intermediate-risk patients using 3-mm PTV margins. At 30 months median follow-up, the 15 patients receiving 45 Gy experienced similar toxicity and efficacy compared to the other SBRT publications. Our observed dose escalation benefit for intermediate- and high-risk patients includes slightly higher late grade III GU toxicity, but no significant difference in GI toxicity. One of the patients with late grade III GU toxicity had the largest prostate volume in the study (3.5 times larger than the median volume), which may have contributed to the severity of his toxicity. While Boike et al. do not report a dose limiting toxicity, their only grade IV event occurred in their highest dose group — a group which had 12 months median follow-up, suggesting this group’s toxicity rate will likely increase. It is also important to note that our treatment is delivered with the CyberKnife, which tracks the fiducial position throughout treatment whereas Boike et al. track fiducials prior to each treatment, not during delivery. As dose increases and PTV margin decreases tracking prostate motion during treatment becomes more important to ensure nearby critical structure dose is limited and target dose is not compromised. Nonetheless, their use of a balloon may substantially limit the movement of the prostate with the risk of higher doses to the anterior rectum.

In the prospective study by King et al. [18], there was a significant reduction in the rate of late grade I toxicities in the subset of patients treated every other day (QOD). There may be a benefit for QOD vs. daily fractionation, however, this phenomenon was not well understood in terms of radiobiology. At the time when our patients were treated, QOD fractions were not used. After analyzing radiobiological models with prominent radiobiologists in late 2008, we have since decided to incorporate at least a 1-day break between treatments, which includes some patients in this study.

Toxicities from IMRT to the prostate have been well documented. Zelefsky et al. [25] report long-term data on late toxicity using an IMRT dose of 81 Gy given in traditional fractionation. At a median follow-up of 7 years with 561 patients, 3 % of patients experienced late grade III GU toxicity (according to the Common Terminology Criteria of Adverse Events toxicity scale) and 9 % had late grade II GU toxicity occurring at a median of 14 months after completion of IMRT. Late GI toxicity manifested in less than 1 % as grade III and 1.5 % as grade II at a median of 13 months after IMRT completion. Our current study shows the same proportion of late grade III GU toxicity and similar late GI toxicity. We roughly compare late toxicity rates despite the difference in length of follow-up between the studies since their median time to late toxicity was 14 months. In a study with similar length of follow-up, Eade et al. report late toxicities in patients treated with IMRT doses of 74–78 Gy or I-125 brachytherapy. Their 3-year actuarial risk of acute and late GU toxicities from IMRT were 1.4 % and 0.5 %, respectively. There were no acute or late grade III GI toxicities [29].

Reported potency preservation rates for SBRT include 40 % at 35 months [28] and 80–82 % at 1 year [16, 17]. Wiegner and King [30] compared ED rates following SBRT to other modalities of radiotherapy alone and concluded ED rates in the proportion of patients receiving SBRT to be similar to the upper end experienced by those receiving other types of monotherapy. In the current study, at a median 31 months 83 % of monotherapy patients remained potent. Importantly, usage of ED medications increases this potency preservation rate to 100 %. Nevertheless, comparison of published potency rates is difficult because different metrics are used and ED medication usage is not uniformly reported.

## Conclusions

These results support the growing body of literature indicating SBRT for prostate cancer is effective and well tolerated with excellent FFBF, PSA nadirs, and acceptable toxicity. The potential benefits of dose escalation in intermediate- and high-risk patients suggest SBRT offers promise for a treatment that has improved convenience, low toxicity, excellent clinical outcomes and likely reduced cost. One limitation of our current study is that BF was not confirmed as local failure by prostate biopsy. This makes it difficult to draw strong conclusions regarding a dose response. We can conclude that dose escalation may be beneficial in specific patient groups, in order to influence future study design. Prospective trials for prostate cancer studying SBRT regimens with long-term follow-up and prostate biopsies to confirm local failure in those with BF are needed to confirm it as an alternative treatment in comparison to IMRT and radical prostatectomy. There is currently a randomized phase II SBRT trial (RTOG 0938) accruing favorable-risk prostate cancer to hypofractionated schedules of 7.25 Gy × 5 and 4.3 Gy × 12. Decisions regarding the optimal treatment will eventually become clearer when data from the randomized trial Prostate Advances in Comparative Evidence compares IMRT to SBRT and SBRT to surgery.
